# The Zinc Transporter Slc30a8/ZnT8 Is Required in a Subpopulation of Pancreatic α-Cells for Hypoglycemia-induced Glucagon Secretion*

**DOI:** 10.1074/jbc.M115.645291

**Published:** 2015-07-15

**Authors:** Antonia Solomou, Gargi Meur, Elisa Bellomo, David J. Hodson, Alejandra Tomas, Stéphanie Migrenne Li, Erwann Philippe, Pedro L. Herrera, Christophe Magnan, Guy A. Rutter

**Affiliations:** From the ‡ Section of Cell Biology and Functional Genomics, Division of Diabetes Endocrinology and Metabolism, Department of Medicine, Imperial College London, London W12 0NN, United Kingdom,; the § Department of Cell Biology, Institute of Ophthalmology, University College London, Greater London EC1V 9EL, United Kingdom,; the ¶ University Paris Diderot-Paris 7, Unit of Functional and Adaptive Biology (BFA) EAC 7059 CNRS, 75013 Paris, France, and; the ‖ Department of Genetic Medicine and Development, Faculty of Medicine, University of Geneva, 1 rue Michel-Servet, 1211 Geneva-4, Switzerland

**Keywords:** calcium, diabetes, glucagon, secretion, zinc

## Abstract

*SLC30A8* encodes a zinc transporter ZnT8 largely restricted to pancreatic islet β- and α-cells, and responsible for zinc accumulation into secretory granules. Although common *SLC30A8* variants, believed to reduce ZnT8 activity, increase type 2 diabetes risk in humans, rare inactivating mutations are protective. To investigate the role of *Slc30a8* in the control of glucagon secretion, *Slc30a8* was inactivated selectively in α-cells by crossing mice with alleles floxed at exon 1 to animals expressing *Cre* recombinase under the pre-proglucagon promoter. Further crossing to Rosa26:tdRFP mice, and sorting of RFP^+^: glucagon^+^ cells from KO mice, revealed recombination in ∼30% of α-cells, of which ∼50% were ZnT8-negative (14 ± 1.8% of all α-cells). Although glucose and insulin tolerance were normal, female αZnT8KO mice required lower glucose infusion rates during hypoglycemic clamps and displayed enhanced glucagon release (*p* < 0.001) *versus* WT mice. Correspondingly, islets isolated from αZnT8KO mice secreted more glucagon at 1 mm glucose, but not 17 mm glucose, than WT controls (*n* = 5; *p* = 0.008). Although the expression of other ZnT family members was unchanged, cytoplasmic (*n* = 4 mice per genotype; *p* < 0.0001) and granular (*n* = 3, *p* < 0.01) free Zn^2+^ levels were significantly lower in KO α-cells *versus* control cells. In response to low glucose, the amplitude and frequency of intracellular Ca^2+^ increases were unchanged in α-cells of αZnT8KO KO mice. ZnT8 is thus important in a subset of α-cells for normal responses to hypoglycemia and acts via Ca^2+^-independent mechanisms.

## Teaser

**Background:** The role of the type 2 diabetes risk gene *SLC30A8*, encoding ZnT8, in the control of glucagon secretion is not clearly established.

**Results:** Inactivation of ZnT8 in a subset of α-cells leads to increased glucagon secretion *in vivo* and *in vitro*.

**Conclusion:** ZnT8 is involved in normal glucagon release.

**Significance:** *SLC30A8* diabetes risk alleles may influence glucagon secretion.

## Introduction

Glucagon is the key counter-regulatory hormone responsible for opposing the glucose-lowering effects of insulin (1). Thus, glucagon stimulates both glycogen breakdown and gluconeogenesis by the liver (2), whereas decreasing hepatic triglyceride synthesis (3, 4). Strikingly, mice deleted for glucagon receptors fail to develop hyperglycemia after the destruction of the β-cell complement (5), demonstrating the likely importance of maintained or increased glucagon signaling in the context of type 2 diabetes (T2D)^4^ (6, 7). By contrast, glucagon secretion is required in type 1 diabetes to ensure an adequate response to insulin-induced hypoglycemia, and a failure of this process contributes significantly to mortality in the latter disease (7). Together, these diseases affect ∼6% of the population worldwide (8).

Recent genome-wide association studies (9–12) have demonstrated that ∼90 loci in the human genome significantly affect the risk of T2D. Although the roles played by the genes in these loci in the control of insulin secretion is under increasing scrutiny using model systems (13, 14), an action to modulate glucagon secretion is, in most cases, unexplored.

The *SLC30A8* gene, encoding the endocrine pancreas-restricted zinc transporter ZnT8, displays one of the strongest effect sizes on T2D risk (∼15% per allele). The risk (thymine) variant at SNP rs13266634 encodes an R325W variant with lower Zn^2+^ transporting activity and thus less able to catalyze the accumulation of Zn^2+^ into insulin-containing granules (15, 16).

Consistent with impaired β-cell function in the absence of ZnT8, we (15, 17) and others (18) have previously shown, using Cre*Lox*P technology, that inactivation of the *Slc30a8* gene in mice, either systemically (15, 17, 18) or selectively in the β-cell (19), leads to abnormal insulin release *in vivo* and impaired glucose tolerance. This is associated with a profound loss of total Zn^2+^ from the β-cell granule and a derangement in the ultrastructure of dense cores, indicative of the failure of insulin to crystallize. Furthermore, recent studies (20) suggest that decreased Zn^2+^ release from the pancreas, and consequently enhanced insulin clearance by the liver, also contributes to lower insulin levels (and an increase in C-peptide/insulin ratio) in carriers of risk variants at *SLC30A8*.

Suggesting that the relationship between variants in *SLC30A8* and diabetes risk may be more complex than previously assumed, rare inactivating mutations in the *SLC30A8* gene have been shown to protect against T2D (21), a result that was unexpected given that inactivation of the gene in mice usually leads to impaired glucose tolerance (see above) (22). This paradox has therefore led us to re-investigate whether there may be a role for ZnT8 in glucagon storage and secretion. Although our earlier studies of the metabolic phenotype of mice in which ZnT8 inactivated selectively in the α-cell did not reveal a marked glycemic phenotype, notably during glucose tolerance tests, the above studies were limited in scope and did not examine the effects of ZnT8 deletion during hypoglycemia (19).

The chief goal of the present work was therefore to re-explore the role of ZnT8 in the control of glucagon secretion and to determine the molecular and cellular basis for any changes identified. We have addressed these questions by combining single cell imaging approaches and *in vivo* analyses of glucose homeostasis in mice lacking the transporter selectively in the α-cell. We show that deletion of ZnT8 in a limited subset (∼15%) of α-cells is sufficient to increase glucagon secretion at low glucose concentrations *in vitro* and *in vivo* and to improve the response to hypoglycemia. Possible mechanisms through which ZnT8 may restrict glucagon release are discussed.

## Experimental Procedures

### 

#### 

##### Animals

Animals were kept in a pathogen-free facility under a 12-h light-dark cycle with access to water and a standard mouse diet (Lillico Biotechnology). The transgenic mouse strains were maintained on a C57/BL6 genetic background. Mice bearing alleles of ZnT8 (Slc30a8) in which exon 1 was flanked by *Lox*P sites were obtained from genOway (Lyon, France). To generate αZnT8 KO mice, ZnT8^fl/fl^ mice (19) were crossed to mice carrying the *Cre* transgene under an ∼0.6-kb fragment of the pre-proglucagon promoter (PPG*Cre*) (23). Breeding pairs were established between PPG*Cre*:ZnT8^fl/fl^ and ZnT8^fl/fl^ mice, allowing comparison between *Cre*+ and *Cre*^−^ littermates. Note that the presence of the PPG*Cre* itself does not impact glycemic phenotype (24) or lead to recombination outside the pancreas (25). For selective labeling of α-cells in *in vitro* experiments, αZnT8 KO mice were further crossed to Rosa26:tdRFP animals. Mice expressing the *Cre* transgene and tdRFP with WT ZnT8 alleles (ZnT8^+/+^:PPG*Cre*^+/−^:RFP^+^) were used as controls in experiments that required α-cell identification (Ca^2+^ and Zn^2+^ imaging). For *in vivo* studies, and *in vitro* experiments using islets that did not require α-cell identification, ZnT8^fl/fl^:PPG*Cre*^−/−^ mice were used as controls. All animal experiments were approved by the UK Home Office under the Animals (Scientific Procedures) Act 1986 (PPL 70/7349).

##### FACS Analysis

Islets were dissociated into single cells as described previously (26), washed in PBS, and centrifuged at 600 × *g* for 2 min. Cells were incubated in 50 μl of near-IR dead cell stain (1:1000; Life Technologies) for 20 min at 4 °C, washed with PBA (PBS, 1% BSA, 0.1% azide), and fixed in 2% paraformaldehyde for 10 min at room temperature. Cells were then washed twice with PBA and once with saponin (0.025% in PBA) before a 10-min incubation with saponin at room temperature. Cells were incubated with primary antibodies against mouse ZnT8 (Mellitech, Grenoble, France) and insulin and glucagon (DAKO and Santa Cruz Biotechnology, respectively) at 1:100 dilution in saponin for 20 min. After two further washes in saponin, cells were incubated with secondary antibodies (anti-mouse Alexa Fluor 405, anti-guinea pig Alexa Fluor 488, anti-rabbit Alexa Fluor 640) for 20 min. Two final washes in saponin were performed before resuspension in PBA. The samples were processed on a BD LSRFortessa flow cytometer (BD Biosciences).

##### Islet Isolation

Islets were isolated essentially as described (27), with minor modifications. Briefly, pancreata inflated with collagenase solution at 1 mg/ml (Serva) were placed in a water bath at 37 °C for 10 min. Cold additive-free RPMI (Sigma) was added followed by centrifugation at 1000 rpm for 1 min. The supernatant was removed before adding ∼15 ml of cold additive-free RPMI medium, and the pancreas was resuspended in the medium. This pellet was washed three more times. The islets were then re-suspended in 3 ml of Histopaque 119 (Sigma). A sucrose gradient was then made by adding dropwise 3 ml of Histopaque 1083 and 1077 (Sigma). Finally, RPMI was added dropwise up to 12 ml. The gradient was centrifuged at 2500 rpm for 20 min. The islets found just below the RPMI layer were collected and centrifuged at 1000 rpm for 1 min after the addition of up to 12 ml of RPMI. The supernatant was removed; islets were re-suspended and put in a Petri dish containing complete RPMI medium. After a few hours of recovery at 37 °C, islets were handpicked and placed in fresh medium.

##### Glucagon Secretion and Extraction of Total Pancreatic Glucagon

Glucagon secretion was measured from groups of 18 size-matched islets per well, incubated for 1 h in 500 μl of Krebs-HEPES bicarbonate buffer at 37 °C and constant agitation supplemented with either 1 mm or 17 mm glucose (26). Total islet glucagon was extracted from islets using acidified ethanol and sonication. Secreted and total glucagon were measured by radioimmunoassay (Millipore). Total pancreatic glucagon was extracted by homogenization in acid ethanol and quantified by HTRF-based (CisBio) assay as described (28).

##### Glucose Tolerance Tests

Mice were fasted overnight before intraperitoneal injection with glucose (1 g/kg). Blood samples were subsequently taken from the tail vein at 0, 15, 30, 60, and 120 min, and glucose concentration was measured with an automatic glucometer (Accucheck, Roche Diagnostics, Burgess Hill, UK).

##### Insulin Tolerance Tests

Mice were fasted for 5 h before intraperitoneal injection with insulin (0.5 units/kg) and blood sampling at 0, 15, 30, and 60 min for glucose concentration as above. Additionally, ∼100 μl of plasma was taken at time 0 and 15 min for glucagon measurements using a radioimmunoassay (Millipore).

##### Hyperinsulinemic/Hypoglycemic Clamp

Mice were injected through a jugular catheter with an insulin bolus (1 units/kg) to decrease glycemia to ∼50 mg/dl within 30–40 min. After this bolus, animals were perfused with insulin at 1.2 units/kg/h for 90 min (3 μl/min). Glucose (20%) was co-perfused with insulin to maintain a plasma glucose level of 50 mg/dl. The concentration of blood glucose was measured every 20 min. Blood was collected before and at the end of the clamp in tubes filled with EGTA (1.6 mg/ml, Sigma) and aprotinin (250 kilo-international units/ml; Sigma) for glucagon measurement.

##### Calcium Imaging

Islets were incubated (37 °C 95% O_2_/5% CO_2_) for 45 min in 10 μm Fluo-2-AM (Invitrogen) dissolved in DMSO (0.01% w/v) and pluronic acid (0.001% w/v) in a bicarbonate buffer containing (in mm) 120 NaCl, 4.8 KCl, 1.25 NaH_2_PO_4_, 24 NaHCO_3_, 2.5 CaCl_2_, 1.2 MgCl_2_, 3 d-glucose). Following incubation, islets were placed in a perifusion chamber, mounted on a Zeiss Axiovert confocal microscope, and perifused continuously at 34–36 °C with buffer containing the indicated glucose concentration. Fluo-2 was excited with a 491-nm laser, and emitted light was filtered at 525/50 nm. Volocity^TM^ software (PerkinElmer) was used for data capture and analysis. Traces are presented as normalized intensity over time (*F*/*F*_min_).

##### Cytosolic Zinc Imaging with FluoZin-3

Islets were incubated (37 °C, 95% O_2_/5% CO_2_) for 1 h in 1 μm FluoZin-3-AM (Invitrogen) dissolved in DMSO (0.01% w/v) in bicarbonate buffer containing 11 mm glucose. Imaging was performed as for Ca^2+^, and results are presented as *F*_1_/*F*_min_ where *F*_1_ = mean baseline fluorescence, and *F*_min_ = minimum fluorescence in the presence of the Zn^2+^ chelator *N*,N*,N*′,*N*′-tetrakis (2-pyridylmethyl) ethylenediamine (TPEN).

##### Granular Zinc Imaging with Zinpyr-4

Islets were incubated as above for 30 min in 10 μm Zinpyr-4 (Santa Cruz Biotechnology). Imaging was performed on a Nikon Ti Eclipse microscope equipped with an X-Light spinning disk and Hamamatsu ORCA-Flash4.0 complementary metal oxide semiconductor detector. Static images were captured at 37 °C using a 63× objective, keeping the exposure time and laser intensity constant throughout. Zinpyr-4 and RFP were excited using 488- and 561-nm lasers, respectively, and signals were monitored at 535/50 nm (Zinpyr-4) and 630/75 nm (RFP). Differences in Zinpyr4 intensity between WT and KO animals were measured using the corrected total cell fluorescence (integrated density − (cell area × mean background fluorescence)), as described (29).

##### Cell Sorting by FACS

After overnight incubation, ∼250–300 islets were washed twice with 5 ml of Hanks'-based cell dissociation buffer (Invitrogen) containing 0.1% BSA, before centrifugation at 2000 rpm for 2 min. The islets were dissociated by repeated pipetting in 150 μl of the solution and 20 μl of trypsin. The reaction was stopped with the addition of 20 μl of FBS (Seralab). Dissociated cells were then washed in 1 ml of PBS, centrifuged, and resuspended in 500 μl of PBS and 1% FBS, before the addition of DAPI (1 μg/ml; Roche Diagnostics) to stain for dead cells. Following a 10-min incubation, cells were filtered through a 35-μm strainer, and RFP^+^ and RFP^−^ cells were recovered in 500-μl TRIzol post-sorting on a BD FACSAria III (BD Biosciences).

##### RNA Extraction

Cells were washed twice in PBS followed by the addition of 500 μl of TRIzol (Invitrogen). Chloroform (200 μl/ml TRIzol) was added, and the tube was inverted 5–6 times and left at room temperature for 5 min until two layers formed. Samples were centrifuged at 12,000 rpm for 15 min at 4 °C. The upper aqueous phase was removed, and 2 μl of glycogen was added before precipitating RNA by adding an equal volume of isopropanol, incubating for 15 min at room temperature, and centrifuging for 15 min at 12,000 rpm. The pellet was washed twice in 1 ml of 75% ethanol and centrifuged at 12,000 rpm for 5 min. The dried pellet was finally resuspended in nuclease-free water.

##### cDNA and RT-PCR

RNA was extracted using TRIzol and cDNA transcribed using a High Capacity reverse transcription kit (Applied Biosystems) according to the manufacturer's instructions. SYBR Green quantitative RT-PCR was performed as described previously (30), using the primer sequences given in Ref. 31 and Table 1.

**TABLE 1:** **Additional primer sequences used for quantitative RT-PCR** FOR, forward; REV, reverse.

ZnT1 FOR	CAGGCAGAGCCAGAAAAATTG
ZnT1 REV	TGGATGAGATTCCCATTTACTTGTAC
ZnT2 FOR	CCCGACCAGCCACCAA
ZnT2 REV	CCAAGGATCTCGGCTCGAT
ZnT3 FOR	GGAGGTGGTTGGTGGGTATTT
ZnT3REV	CAAGTGGGCGGCATCAGT
ZnT4 FOR	CATCGCTGCCGTCCTCTAC
ZnT4 REV	ATTTGCCATGTATCCACCTACAAG
ZnT5 FOR	ACTGTTTGCTGCCCTGATGAG
ZnT5 REV	CTCTATTCGGCCATACCCATAGG
ZnT6 FOR	ACGTCTCTGAAGCTGCTAGTACGA
ZnT6 REV	CCACACAAGCTGCGGCTAA
ZnT7 FOR	TGTGCCTGAACCTCTCTTTCG
ZnT7 REV	GCCTAGGCAGTTGCTCCAGAT
ZnT8 FOR	TGCACAGTCTACACATCTGGTCACT
ZnT8 REV	TGGCTGGCAGCTGTAGCA
ZnT9 FOR	GCACTGGGCATCAGCAAAT
ZnT9 REV	GAAAAGCCGTACGGGTGAGA
MT1 FOR	CCTTCTCCTCACTTACTCCGTAGC
MT1 REV	GGAGCCGCCGGTGGA
MT2 FOR	TCCTGTGCCTCCGATGGAT
MT2 REV	TGCAGGAAGTACATTTGCATTGT
MT3 FOR	AAGTGCAAGGGCTGCAAATG
MT3 REV	CACAGTCCTTGGCACACTTCTC
Actin FOR	CGAGTCGCGTCCACCC
Actin REV	CATCCATGGCGAACTGGTG

##### Immunostaining

Pancreata were fixed in 10% formalin overnight at 4 °C, embedded in wax, and sliced at 5 μm. Sections were then dewaxed using Histoclear (Sigma) and rehydrated by washing in 100, 95, and 70% ethanol and distilled water. The sections were treated with antigen unmasking solution (Vector Laboratories), before washing in PBS and blocking in PBS containing 0.1% Triton X-100, 2% BSA, and 2% goat and donkey serum for 60 min at room temperature. Primary antibodies (anti-guinea pig insulin (Dako), 1:200, anti-rabbit glucagon (Sigma), 1:100) in PBS were then applied overnight at 4 °C. The next day, slides were washed three times in PBS, before incubation with secondary antibodies (Alexa Fluor 488 goat anti-guinea pig 1:1000, Alexa Fluor 568 donkey anti-rabbit 1:500) in PBS at room temperature, and mounting using VECTASHIELD with DAPI. Imaging was performed using a Zeiss Axio Observer widefield microscope using a 40× objective. The α:β-cell ratio was determined by subjecting insulin and glucagon immunopositive regions to manual thresholding (ImageJ macro) and dividing the α-cell area by the β-cell area. For analysis of the subcellular distribution of ZnT8 and glucagon, islets were dissociated into single cells with trypsin (Hanks' buffer, Invitrogen, Paisley, UK) and, after fixation in 4% (v/v) paraformaldehyde and permeabilization with Triton X-100 as above, treated with anti-ZnT8 (1:200; Mellitech) and anti-glucagon (1:1,000) antibodies, in PBS containing 1% (w/v) BSA before mounting. Images were captured with a Zeiss Axiovert confocal microscope (32) using a 63× objective.

##### Statistical Analysis

Student's *t* test was used to identify the significance of any difference between two independent variables. Interactions between multiple treatments were determined using two-way ANOVA (adjusted for repeated measures as appropriate), followed by pairwise comparisons with Bonferroni's post hoc test. All analyses were performed using GraphPad Prism 6.0, and results were considered significant at *p* < 0.05. Values are presented as means ± S.E.

## Results

### 

#### 

##### Recombination and Deletion of ZnT8 in the α-Cell

**FIGURE 1.**
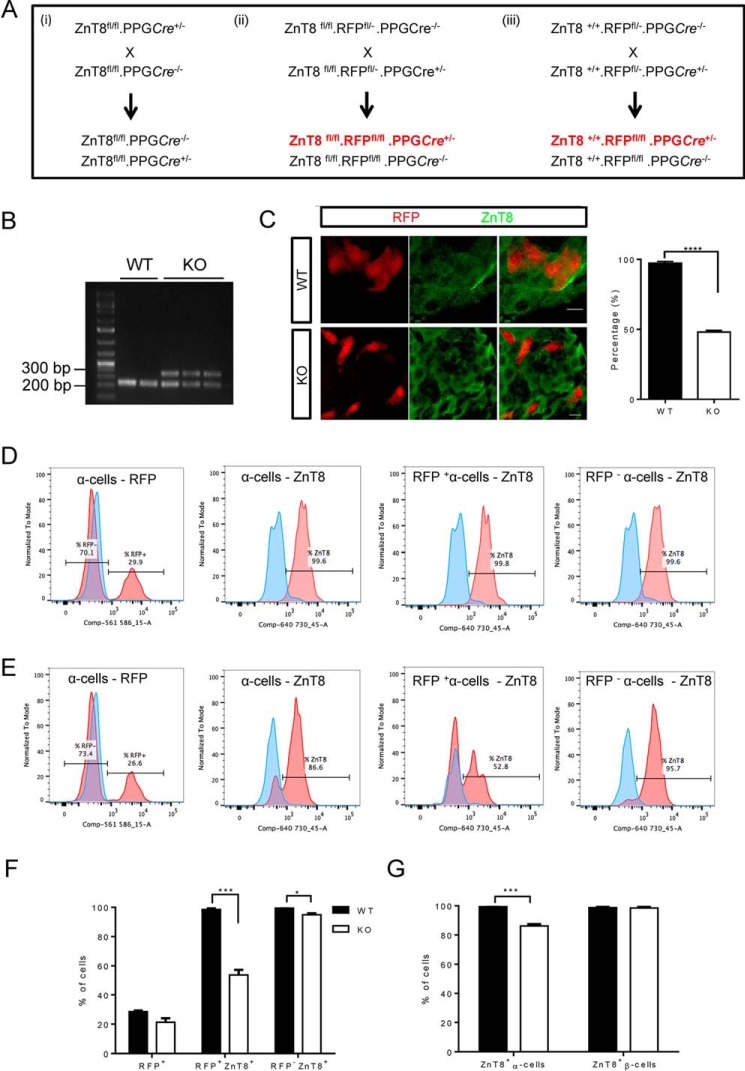
**ZnT8 deletion in α cells and assessment of recombination.** *A*, breeding strategy used to generate αZnT8 KO mice and respective controls: (*i*) for *in vivo* comparisons requiring littermate comparisons but not requiring RFP labeling; (*ii*) to produce RFP-labeled, ZnT8 null α-cells; and (*iii*) to produce RFP-labeled α-cells with WT ZnT8 alleles. *B*, PCR genotyping of WT and null animals. The bands at ∼330 bp were amplified from the *Cre* transgene, and the bands at ∼220 bp were amplified from β-catenin, used as a PCR control. *C*, ZnT8 immunoreactivity assessed in islets from WT and αZnT8 KO mice. Rabbit anti-ZnT8 antibody (Mellitech) was added at 1:200 dilution and subsequently revealed with a secondary anti-mouse IgG antibody conjugated to Alexa Fluor 488. Representative images thus show ZnT8 in *green* and endogenous RFP fluorescence in *red. Scale bar* = 10 μm. The histogram shows the percentage of cells positive for both RFP and ZnT8. *n* = 80–90 cells from 5–6 islets per genotype. ****, *p* < 0.0001 by Student's *t* test. *D* and *E*, single cells from RFP^+^ WT and KO islets were fixed, permeabilized, and stained for glucagon, insulin, and ZnT8 (see “Experimental Procedures”). Cells alive immediately before fixation were gated based on their exclusion of a dead cell marker and analyzed according to the expression of the proteins stained. *D* and *E*, representative traces showing expression analysis for WT (*D*) and KO (*E*). *Red traces* reflect the positive populations, and *blue traces* reflect the control-negative populations. *F*, expression of RFP and ZnT8 in WT and KO α-cells as a percentage of live cells. *G*, ZnT8 expression in glucagon^+^ and insulin^+^ cells from WT and KO mice. Data are mean ± S.E., *n* = 3, two-way ANOVA, *, *p* < 0.01, ***, *p* < 0.0005.

To investigate the role of ZnT8 in the α-cell, we generated and studied mice deleted selectively in the α-cell for ZnT8. Inactivation was achieved using *Cre* recombinase under the control of the pre-proglucagon promoter (PPG*Cre*) (23) to delete the first exon of the *Slc30a8* gene. For *in vivo* studies, littermate controls were used as generated under Fig. 1*A* (*i*). To allow identification of α-cells in these islets in *in vitro* experiments, the resulting mice were further crossed with animals bearing tdRFP 3′ to a stop-flox-stop cassette at the Rosa26 locus (Rosa26tdRFP; Fig. 1*A* (*ii*)). A separate colony of animals, WT for the *Slc30a8* locus, was bred bearing the Rosa26:tdRFP transgene and PPG*Cre*, for use as controls in *in vitro* experiments (Fig. 1*A* (*iii*)). PCR amplification of DNA extracted from ear biopsies was performed to determine genotype (*e.g.* Fig. 1*B*). There were no apparent differences in growth and body weight between WT and KO animals (not shown).

We have previously reported (33), using islets from RFP^+^PPG*Cre*^+/−^ mice and staining for RFP and glucagon or insulin, that ∼45% of glucagon-positive cells expressed RFP, whereas a negligible (∼2%) proportion of insulin-positive cells also expressed RFP. Here, we used immunocytochemical analysis of whole islets to reveal that ZnT8 immunoreactivity was selectively reduced in the RFP^+^ population in PPG*Cre*:ZnT8^fl/fl^ but not WT mice (Fig. 1*C*). To more accurately quantify the degree of deletion of the *Slc30a8* allele, RFP-positive islets from WT and KO mice were dissociated and stained for insulin, glucagon, and ZnT8 before analysis by flow cytometry (Fig. 1, *D* and *E*). Examined in both WT and KO mice, ∼30% of glucagon-positive cells were also positive for RFP. Although ZnT8 was expressed in ∼99.7% of RFP^+^ cells in the WT, there was an ∼50% (48 ± 5%) reduction in immunodetectable ZnT8 in KO RFP^+^ cells. Additionally, we observed a 5–6% reduction of ZnT8 expression in glucagon^+^ RFP^−^ cells (Fig. 1*F*). Considering the whole population of α-cells, the overall decrease in ZnT8 was about 14% (14 ± 1.8%), whereas no deletion was detected in the β-cell population (Fig. 1*G*). Hence, PPG*Cre* deletes ZnT8 highly specifically, but only in a minority of α-cells, consistent with previous results (33).

##### Normal Glucose Homeostasis and Insulin Sensitivity but Enhanced Response to Hypoglycemia in Vivo and Low Glucose in Vitro

**FIGURE 2.**
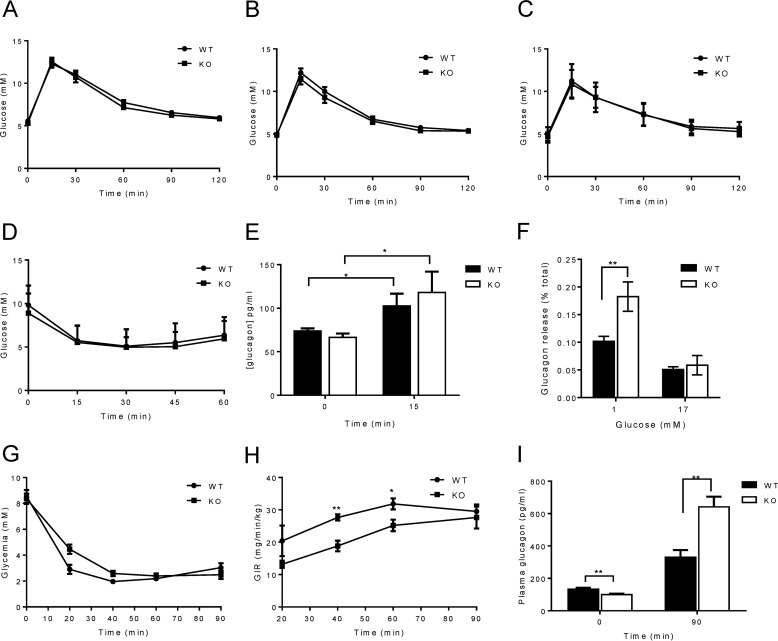
**Glucose homeostasis and responses to hypoglycemia.** *A–C*, intraperitoneal glucose tolerance tests were performed on WT and αZnT8 KO mice fed a normal chow diet after an overnight fast at 8 (*A*), 12 (*B*), and 16 (*C*) weeks of age. *n* = 8–15 mice/genotype. *D*, intraperitoneal insulin tolerance tests performed on 8-week-old mice fasted for 5 h and injected with 0.5 units/kg of insulin. *E*, plasma glucagon measured at 0 and 15 min. *n* = 13–14 mice/genotype. *F*, glucose-inhibited glucagon secretion from WT and αZnT8 KO islets incubated with high (17 mm) and low (1 mm) glucose. *G*, hypoglycemic hyperinsulinemic clamps were performed on 12-week-old mice. *H*, glucose levels were monitored, and glucose infusion rate were adjusted accordingly. *I*, plasma glucagon levels at 0 and 15 min. *n* = 4–6 mice/genotype. Data are mean ± S.E., two-way repeated measures ANOVA or two-way ANOVA. *, *p* < 0.05, **, *p* < 0.01.

Intraperitoneal glucose tolerance tests were performed on WT and KO mice at the ages of 8, 10, and 12 weeks (Fig. 2, *A–C*), to assess whether the absence of ZnT8 affects glucose metabolism. Glucose clearance was very similar in WT and KO mice across all the ages examined. In an effort to exert some extra stress on the α-cell and to check whether deletion of ZnT8 affects insulin sensitivity, insulin tolerance tests were performed on mice at 8 weeks of age. Blood glucose was measured at regular intervals and, similarly to intraperitoneal glucose tolerance tests, there were no significant differences between WT and KO mice (Fig. 2*D*). Blood was collected at 0 and 15 min for plasma glucagon measurements. The glucagon levels of WT and KO mice were similar at both time points (Fig. 2*E*).

To assess whether α-cell ZnT8 KO mice respond differently under stimulatory conditions, glucagon secretion experiments were performed on isolated WT and KO islets incubated with 1 or 17 mm glucose. At 17 mm glucose, glucagon secretion was similar between genotypes, whereas at the stimulatory concentration (1 mm glucose), KO islets secreted significantly more glucagon than islets from WT controls when normalized to glucagon content per islet (Fig. 2*F*). No differences in total pancreatic glucagon content were apparent between WT and KO mice (1.6 ± 0.35 and 1.4 ± 0.1 ng/mg of pancreas, *n* = 3 mice/genotype).

Additionally, WT and KO mice were examined using hypoglycemic clamps. During this procedure, mice were rendered hypoglycemic with the administration of insulin (Fig. 2*G*) and were then maintained in a steady, mild hypoglycemic state by controlling the rate of glucose infusion. Female αZnT8 KO mice required a lower glucose infusion rate than WT controls to maintain the same glycemia (Fig. 2*H*), implying that they secreted more glucagon than WT controls. Blood samples taken from these mice showed that, at time 0, KO mice had a lower basal secretion, but at 90 min after the imposition of hypoglycemia, they secreted significantly more glucagon than WT controls (Fig. 2*I*).

##### Altered Intracellular Free Zn^2+^ levels, but Unchanged Intracellular Ca^2+^ Responses to Glucose Deprivation, after ZnT8 Deletion

**FIGURE 3.**
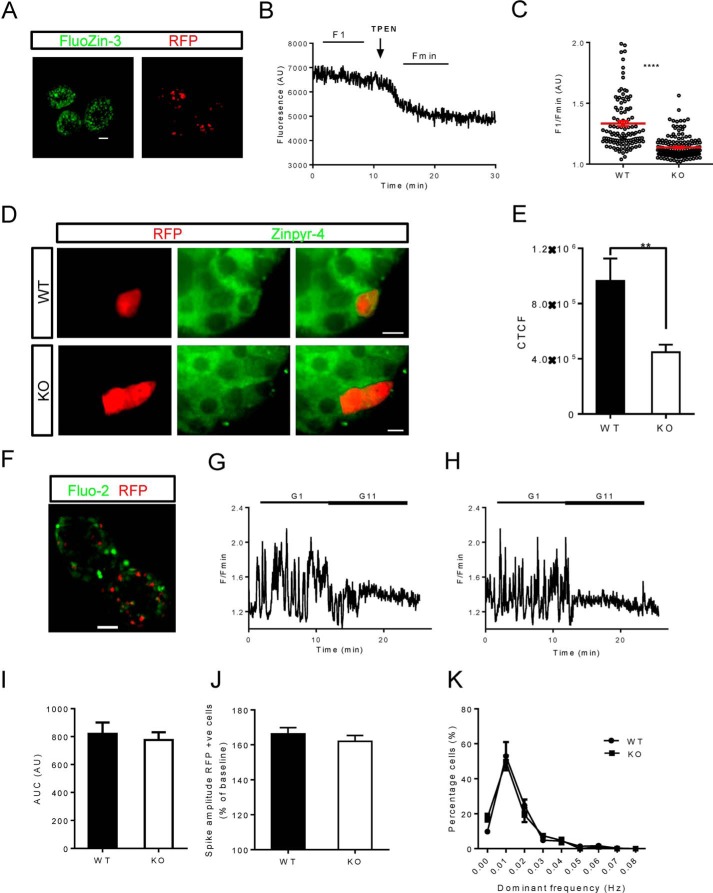
**Intracellular free Zn^2+^ and Ca^2+^ imaging in RFP^+^ WT and αZnT8KO islets.** *A*, the cell-permeable dye FluoZin-3 was used for Zn^2+^ imaging. *Scale bar* = 50 μm. *B*, islets were imaged at baseline (*F*_1_) and after perifusion with TPEN (*F*_min_) to chelate intracellular Zn^2+^. *C*, Zn^2+^ levels were determined from the relative fluorescence (*F*_1_/*F*_min_). *AU*, absorbance units. *D*, imaging of intragranular Zn^2+^ with Zinpyr-4 in WT or KO islets as indicated. *E*, quantification of the changes shown in *D. CTCF*, corrected total cell fluorescence. *n* = 15–20 islets from three mice/genotype, Student's *t* test, **, *p* < 0.01. *F–H*, for Ca^2+^ imaging, islets were incubated for 40 min. with Fluo2 (*F*) before being imaged at low (1.0 mm) and then high (11 mm) glucose. *Scale bar* = 50 μm. *G* and *H*, representative traces from WT (*G*) and KO (*H*) α-cells. *I*, area under the curve (*AUC*). *J*, spike amplitude as a percentage of baseline. +*ve cells*, positive cells. *K*, fast Fourier transform of individual Ca^2+^ traces. Data are mean ± S.E., *n* = 16–20 islets from four mice, Student's *t* test, ***, *p* < 0.0001

To assess whether deletion of ZnT8 affected the levels of cytoplasmic free Zn^2+^, RFP^+^ WT and KO islets were loaded with the zinc indicator FluoZin-3 (Fig. 3*A*). Whole islets were imaged at baseline and after the addition of TPEN (Fig. 3*B*). These two values were used to give an indication of intracellular free Zn^2+^ concentration. In KO α-cells, this value was significantly reduced as compared with those from WT controls (Fig. 3*C*).

Given the probable role of ZnT8 to maintain granular zinc concentrations, we next measured the latter parameter using the granule-tropic fluorescent Zn^2+^ sensor, Zinpyr-4 (34) (Fig. 3*D*). As compared with RFP^+^ cells in WT islets, those from KO animals displayed an ∼50% decrease in apparent free granule Zn^2+^ (Fig. 3*E*). We note that the above changes in intracellular Zn^2+^ distribution are likely to reflect an underestimate of the change in ZnT8 null α-cells, which represent approximately half of the RFP^+^ population (Fig. 1, *C–G*).

To determine whether changes in intracellular free calcium ([Ca^2+^]*_i_*) responses could account for the differences observed in glucagon secretion, whole islets were incubated with the trappable intracellular Ca^2+^ indicator Fluo-2 (Fig. 3*F*). Images were then captured during perifusion at 1 or 17 mm glucose (Fig. 3, *G* and *H*). When analyzed, spike amplitude and area under the curve (Fig. 3, *I* and *J*) were, however, similar in WT and KO α-cells. Moreover, fast Fourier transform of the individual traces showed that WT and KO α-cells oscillated at similar frequencies (Fig. 3*K*).

##### α-Cell Mass and Gene Expression

**FIGURE 4.**
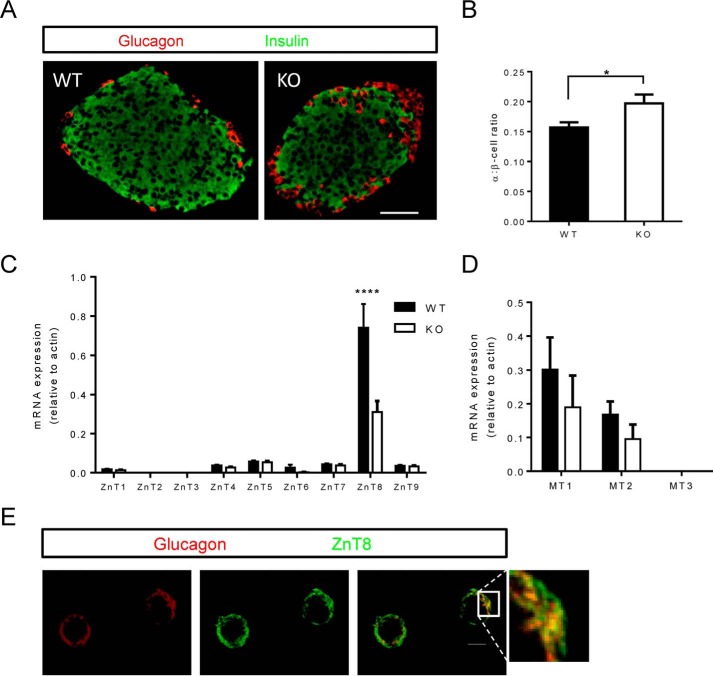
**Pancreatic histology.** *A*, pancreatic slices from WT and ZnT8 KO mice were immunostained for insulin and glucagon. *B*, area is shown as α:β-cell ratio. *Scale bar* = 50 μm. *C* and *D*, FACS-sorted RFP^+^ cells were used for gene expression analysis. *C* and *D*, expression levels of the ZnT family of transporters (*C*) and metallothioneins (*D*) in WT and KO cells. Data are mean ± S.E., *n* = 5–6 mice/genotype, comparisons by two-way ANOVA. *, *p* < 0.05, ****, *p* < 0.0001. Note that in *C* the apparent change in ZnT6 expression was not statistically significant (*p* > 0.9). *E*, assessment of colocalization between ZnT8 and glucagon immunoreactivity in dissociated wild type α-cells. *Scale bar* = 10 μm. See the legend for Fig. 1 and “Experimental Procedures” for further details.

An increase in pancreatic α-cell mass, or a change in the proportion of α-to-β-cells, could conceivably contribute to the increased glucagon secretion observed in KO islets. To investigate this, pancreatic slices were immunostained for glucagon and insulin and imaged to establish the α- and β-cell area (Fig. 4*A*). In KO mice, there was a tendency for increased α-cell area as compared with the α- to β-cell ratio of control mice (Fig. 4*B*).

RFP^+^ WT or KO cells were FACS-sorted for RNA extraction and gene expression analyses. Similar to the results from flow cytometry analysis, we observed in KO cells an ∼50% decrease in ZnT8 expression (Fig. 4*C*). Importantly, there was no compensatory change in the expression of ZnT transporters in the absence of ZnT8, which might have explained the observed lower intracellular free Zn^2+^ levels (Fig. 4*C*). The expression of metallothioneins, cytoplasmic zinc-binding proteins, was likewise comparable in each genotype, although a tendency was apparent for a decrease in MT1 and MT2 expression, in line with the lowered cytosolic free Zn^2+^ levels (Fig. 4*D*).

##### Subcellular Localization of ZnT8 in α-Cells

Our previous studies (15) suggested that ZnT8 was only partially localized to glucagon secretory granules. Extending these findings, we observed here using confocal microscopy (Fig. 4*E*) extensive, although not complete (Pearson correlation = 0.79) overlap between ZnT8 and glucagon immunoreactivity.

## Discussion

The principal aim of the present study was to determine whether ZnT8 plays a role in the control of glucagon secretion. Our findings demonstrate a clear role for ZnT8 in restricting glucagon release at low glucose concentrations both *in vivo* and *in vitro*. Remarkably, deletion of ZnT8 from only a minor fraction (∼15%) of α-cells was sufficient to potentiate the release of the hormone, suggesting either that the effects at the level of individual α-cells are much larger or, alternatively, that the deleted cells play a particularly important role in the activity of the remaining (undeleted) α-cells within the islet (36). Although the present data do not exclude either possibility, we believe that the latter is the likelier given the augmentation in secretion required from individual cells to explain the overall increase in secretion (∼7-fold in the 15% of recombined cells to achieve a 2-fold change).

### 

#### 

##### Role of ZnT8 in the Control of Cytosolic and Granular Free Zn^2+^ Concentration

Measurements of cytosolic Zn^2+^ were confined here to the use of the small chemical probe, FluoZin-3, which indicated significant decreases in this parameter after ZnT8 deletion. We would note that because ZnT8 was only deleted in about 50% of RFP^+^ cells (Fig. 1, *C–G*), our measurements are likely to underestimate the decrease in free Zn^2+^ engendered by ZnT8 suppression in α-cells. Although we were able to distinguish between signals in α-cells from KO *versus* WT islets, we were unable to calibrate these precisely due to the dynamic range (minimum to maximum) of the probe, which even surpassed that of our highly sensitive 16-bit EM-CCD detector (*i.e.* 16^6^ = 65,536 possible gray values), as also independently noted in Ref. 37. Given the *K_d_* of this probe for Zn^2+^ (15 nm at pH 7.4), this observation is nonetheless compatible with free Zn^2+^ concentration in WT cells of close to or just below 1 nm, which was further lowered by ZnT8 deletion. In this respect, the effects of ZnT8 deletion on free Zn^2+^ appear to be similar in α- as in β-cells (38), and suggest a paradoxical role for ZnT8 in the *efflux* as well as the uptake of Zn^2+^ into the secretory granule, the latter evidenced by a clear decrease in granular free Zn^2+^ in αZnT8 KO mouse α-cells (Fig. 3, *D* and *E*). The observed changes in cytosolic and granular Zn^2+^ do not appear to involve alterations in the expression of other ZnT family members, or metallothioneins, although we cannot exclude a possible contribution from changes in Zip (*Slc39a*) Zn^2+^ importer levels. Although attempts were made to use more sophisticated, recombinant, and subcellularly targeted ratiometric biosensors for Zn^2+^ including eCALWY4 (37), we observed very poor expression of this probe in α-cells after viral transduction, in-line with the previously observed tropism of adenoviruses for β-cells within the islet (39, 40).

##### Possible Molecular Mechanisms Involved in the Effects of ZnT8 on Glucagon Secretion

We noted a small (∼15%) increase in α-cell:β-cell ratio in ZnT8 null mice (Fig. 4*B*), which might reflect an action of Zn^2+^ to inhibit cellular proliferation (41). However, we suspect that the quantitatively more important action of ZnT8 is to restrict the release of glucagon stimulated by low glucose concentrations *in vitro* (Fig. 2*F*) and *in vivo* (Fig. 2*I*). There are a number of plausible mechanisms through which ZnT8 might inhibit glucagon secretion at low glucose. Firstly, it is possible that an action via impaired Zn^2+^ release from α-cells is involved. Our previous studies (17) demonstrated that ablation of ZnT8 from β-cells eliminates the co-release of Zn^2+^ alongside insulin, and we can predict that a similar suppression of Zn^2+^ production occurs from the α-cell after ZnT8 deletion. Could this lead to a lowering of Zn^2+^ levels locally, with consequences for glucagon secretion? For example, this might inhibit K_ATP_ channels, reported to be activated by Zn^2+^ in rat (42) but not mouse (43) α-cells to de-inhibit electrical activity and hence provoke the Ca^2+^ oscillations that drive secretion. In the rat, convincing evidence was provided for an inhibitory role for Zn^2+^, based on the ability of the Zn^2+^ chelator Ca^2+^-EDTA to enhance glucagon secretion from the perfused pancreas (44). On the other hand, our own laboratory failed to detect any effects of exogenously applied Zn^2+^ on glucagon release from mouse islets (26). Wheeler and colleagues (45) reported that levels of Zn^2+^ above 10 μm inhibited glucagon secretion by a mechanism not involving K_ATP_ channels, but requiring uptake into the cell and an undefined cytosolic action of Zn^2+^. Nonetheless, and arguing against a role for changes in Zn^2+^ secretion, we did not observe in the present studies any detectable changes in Ca^2+^ dynamics in RFP^+^ cells from αZnT8 KO mice (where ∼50% were ZnT8 null). Although we cannot exclude a small change in Ca^2+^ dynamics that was too small to be detected in the combined (ZnT8^+^, ZnT8^−^) population of RFP^+^ cells, we note that changes in free cytosolic (Fig. 3, *A–C*) and granular (Fig. 3, *D* and *E*) free Zn^2+^ were readily detected between these two groups (RFP^+^ and RFP^−^). Furthermore, we obtained no evidence for the existence of two pools of RFP^+^ cells with differing Ca^2+^ spike frequency (results not shown). Indeed, data from both KO and WT islets passed the D'Agostino Pearson omnibus normality test, suggestive of Gaussian distributions.

Might an effect on glucagon packing within granules be involved? Although we did not see marked abnormalities in glucagon packing by electron microscopy (results not shown), it is conceivable that a “looser” agglomeration of glucagon monomers within the granule facilitates the release of the hormone through the dilating fusion pore, and may mean that a greater proportion of exocytotic events proceed to completion (*i.e.* the granule is fully depleted) before membrane recapture (which may occur rapidly after release through a “kiss and run” process) (46). Our results do, nonetheless, provide clear evidence for a lowering of cytosolic free Zn^2+^, a change likely to exert multiple effects on cellular function and exocytosis, ranging from changes in phosphodiesterase activity, and hence intracellular cAMP levels, to altered signaling through receptor phosphotyrosine phosphatases (47).

##### Relevance for the Understanding of the Effect of SLC30A8 Alleles on T2D Risk

Recent results from Flannick *et al.* (21) have provided a conundrum in terms of our understanding of the role of *SLC30A8*/ZnT8 in the control of hormone secretion and consequently disease risk. As discussed (22), this complexity may arise in part from the interplay between pancreas and liver, because secreted Zn^2+^ apparently plays an important role in controlling insulin clearance by the latter (20). The present results provide a further level of complexity by showing that ZnT8 expression in the islet may affect the subsequent metabolism (clearance) of both glucagon and insulin. However, although the control by Zn^2+^ of glucagon clearance by the liver is a theoretical possibility, the amounts of Zn^2+^ released by α-cells are expected to be far lower than those from β-cells (∼20%, considering the ratio of α- to β-cells in the pancreas (48), and presuming similar maximal rates of release of each hormone).

Corroborating earlier findings (35), we demonstrate partial colocalization between ZnT8 and glucagon immunoreactivity in α-cells (Fig. 4*E*). Interestingly, the degree of colocalization was somewhat lower than that observed in β-cells in our earlier studies. The identity of the non-dense core granule structures on which ZnT8 resides in the α-cell must at this stage remain speculative, but may, in part, represent immature granules.

Interestingly, recent data from human islet samples indicate a strong correlation between the expression of *SLC30A8* and proglucagon in islets, and thus suggest a role for ZnT8 in the control of proglucagon expression, or vice versa (49). Moreover, *SLC30A8* is a target for transcription factor 7-like 2 (TCF7L2) (49), variants of which have the highest impact on disease risk all of the T2D risk genes discovered so far by genome-wide association (14). Thus, ZnT8 may be part of a regulatory network of diabetes susceptibility genes (35). Variants in these genes may impact both β-cell and α-cell function to influence insulin:glucagon ratios and hence T2D risk.

## Author Contributions

G. A. R. conceived and coordinated the study and wrote the paper with A. S. and D. J. H. A. S. performed experiments and analyzed data in Figs. 1–4. S. M. L., G. M., E. B., E. P., and C. M. performed the experiments and analyzed the data shown in Fig. 2, *D–I*. D. J. H. and A. S. performed the experiments shown in Fig. 3. P. H. generated and provided the PPGCre mouse line. All authors reviewed the results and approved the final version of the manuscript.

## Footnotes

4 The abbreviations used are:

T2D: type 2 diabetes
PPG: proglucagon
tdRFP: tandem dimer RFP
ZnT: zinc transporter
DMSO: dimethyl sulfoxide
TPEN: *N*,N*,N*′,*N*′-tetrakis (2-pyridylmethyl) ethylenediamine
ANOVA: analysis of variance.
